# The *MAPT* p.A152T variant is a risk factor associated with tauopathies with atypical clinical and neuropathological features

**DOI:** 10.1016/j.neurobiolaging.2012.04.006

**Published:** 2012-09

**Authors:** Eleanna Kara, Helen Ling, Alan M. Pittman, Karen Shaw, Rohan de Silva, Roberto Simone, Janice L. Holton, Jason D. Warren, Jonathan D. Rohrer, Georgia Xiromerisiou, Andrew Lees, John Hardy, Henry Houlden, Tamas Revesz

**Affiliations:** a Reta Lila Weston Laboratories and Department of Molecular Neuroscience, UCL Institute of Neurology, Queen Square, London, UK; b Department of Neurodegenerative Disease, Dementia Research Centre, UCL Institute of Neurology, University College London, Queen Square, London, UK

**Keywords:** MAPT, Parkinsonism, Corticobasal degeneration, Genetics, Postencephalitic parkinsonism

## Abstract

Microtubule-associated protein tau *(MAPT)* mutations have been shown to underlie frontotemporal dementia and a variety of additional sporadic tauopathies. We identified a rare p.A152T variant in *MAPT* exon 7 in two (of eight) patients with clinical presentation of parkinsonism and postmortem finding of neurofibrillary tangle pathology. Two siblings of one patient also carried the p.A152T variant, and both have progressive cognitive impairment. Further screening identified the variant in two other cases: one with pathologically confirmed corticobasal degeneration and another with the diagnosis of Parkinson's disease with dementia. The balance of evidence suggests this variant is associated with disease, but the very varied phenotype of the cases with the mutation is not consistent with it being a fully penetrant pathogenic mutation. Interestingly, this variation results in the creation of a new phosphorylation site that could cause reduced microtubule binding. We suggest that the A152T variant is a risk factor associated with the development of atypical neurodegenerative conditions with abnormal tau accumulation.

## 1 Introduction

Tau belongs to the family of the microtubule-binding proteins (*MT*) (Witman et al., 1976) and is encoded by the MT-associated protein tau (*MAPT*) gene located on chromosome 17 (17q21) (Andreadis et al., 1992). *MAPT* mutations have been linked to a variety of neurodegenerative diseases with abnormal tau accumulation, and mainly to frontotemporal dementia (FTD) (Hutton et al., 1998) but also to other sporadic tauopathies, including progressive supranuclear palsy (PSP), corticobasal degeneration (CBD), and various disorders with more unusual tau pathology (Conrad et al., 1997; Höglinger et al., 2011; Momeni et al., 2009). Recently, an A152T variation in *MAPT* exon 7 was identified in a patient who had dementia and unclassifiable tauopathy (Kovacs et al., 2011). With this background, we determined to sequence the *MAPT* gene in our small series of cases with indeterminate tauopathies (eight cases). Here, we report two patients with atypical parkinsonian disorder and abnormal tau accumulation at postmortem, both of whom were identified to carry the A152T variation. We then screened a larger series of tauopathy cases for this mutation (PSP and CBD) and some cases with idiopathic Parkinson's disease to determine the nature of the phenotype that seemed to be associated with this variant and found an additional case with CBD as well as a case of Parkinson's disease in which there was prominent tangle pathology.

## 2 Methods

All subjects in this report had provided written consent to perform neuropathological and genetic studies. The access to clinical records and pathological material at the Queen Square Brain Bank (QSBB) has generic ethical approval from a London Multi-Centre Research Ethics Committee under a license from the Human Tissue Authority.

### 2.1 Neuropathology

After postmortem the brains were divided midsagittally. One half brain was immediately frozen and stored at −80 °C, whereas the other half was immersed and fixed in 10% neutral formalin for 3 weeks. Tissue blocks were processed using standard protocols. We performed hematoxylin and eosin, Luxol fast blue/cresyl violet, Congo red staining on 7-μm-thick sections and also used the modified Bielschowsky and Gallyas silver impregnation methods. Immunohistochemistry with antibodies to phospho-tau (AT8 clone recognizing Ser202/Thr205), 3-repeat (3R) and 4-repeat (4R) tau isoforms (de Silva et al., 2003), ubiquitin, p62, TAR DNA-binding protein-43 (TDP-43), and α-synuclein was also carried out using a standard avidin-biotin method.

### 2.2 Patients from unclassified tauopathy series

In our initial DNA-sequencing study, we sequenced the entire *MAPT* gene open reading frame in eight cases that had received a clinical diagnosis of postencephalitic parkinsonism (PEP) or clinical parkinsonism with unclassifiable tauopathy at postmortem. PEP is a rare clinical entity characterized by the development of parkinsonism after the development of encephalitis (Geddes et al., 1993). Initial postmortem studies indicated PEP in case 1, described later in the article, and was previously reported as such (Geddes et al., 1993). The original neuropathological investigation did not reach a conclusive diagnosis in case 2, which had been categorized as “parkinsonism associated with unclassifiable neurofibrillary tangle pathology”.

### 2.3 Genetic analysis

Genomic DNA was extracted from brain tissue of the eight archival cases. In these cases, the *PARK2* and *LRRK2* genes had also been previously fully sequenced without finding any changes. Three of these eight cases have been previously reported (case 5, 7, and 8) (Geddes et al., 1993). After the p.A152T mutation was found in exon 7 in two of these cases, additional screening of this exon was carried out in blood-derived DNA of the three siblings of case 1 (two suffering from dementing illnesses and one unaffected), and in 150 neuropathologically defined control subjects and 133 1958 Wellcome Trust blood donor control subjects. At this stage, the occurrence and frequency of this variant in public databases were also assessed.

### 2.4 Statistical analysis

Fisher exact tests were conducted using an online tool research.microsoft.com/enus/um/redmond/projects/mscompbio/FisherExactTest/. A *p*-value less than 0.01 was considered statistically significant.

### 2.5 Secondary screening

After we had identified the variant in the unclassified tauopathy cases described earlier and failed to find the variant in control subjects, we screened for the mutation by sequencing *MAPT* exon 7 in DNA derived from the brains of the QSBB archival collection, including 114 cases with PSP (Pittman et al., 2004), 8 cases with CBD (Houlden et al., 2001), and 48 cases with idiopathic Parkinson's disease, all of which had received pathological confirmation of their diagnoses.

## 3 Results

### 3.1 Primary sequencing

#### 3.1.1 Genetics

**Fig. 1:**
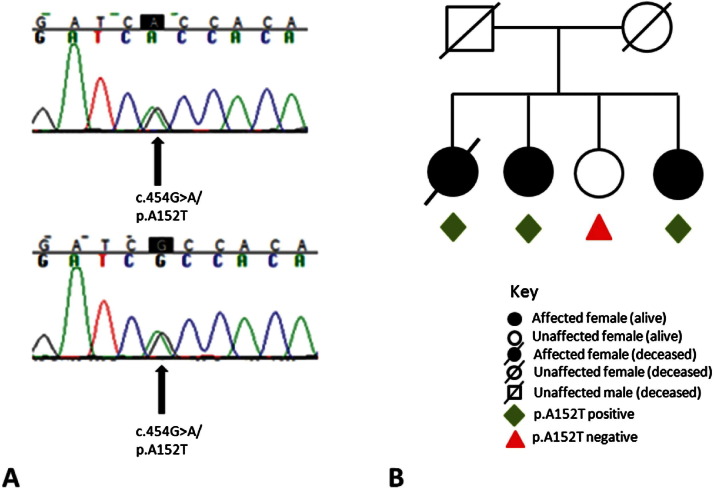
(A) Sequencing chromatograms. c.454G>A/p.A152T variant in patient 1 (upper panel) and patient 2 (lower panel). (B) Family tree of patient 2 showing segregation of the variant with the disease. For interpretation of the references to color in this figure legend, the reader is referred to the Web version of this article.

Among the eight cases subjected to primary genetic analysis, two cases (25%) reported earlier were identified to carry a heterozygote nonsynonymous variant in exon 7 (rs143624519, c.454G<A, p.A152T) (accession numbers NM_005910.5 and NP_005901.2, respectively) (Fig. 1A and 1B). This variant was also found in the two sisters of case 1, who are both developing progressive cognitive impairment, but this variant was absent in the third unaffected sister. All patients are Caucasian and of Northern European descent. This variant is present with a frequency of 19 in 3510 (0.54%) individuals of European American origin (19 in 7020 alleles) in the publically available database from the National Heart, Lung, and Blood Institute (NHLBI) “Grand Opportunity” Exome Sequencing Project (GO-ESP) (Exome Variant Server, NHLBI ESP, Seattle, WA [URL: evs.gs.washington.edu/EVS/] [accessed on 01/2012]). We additionally screened 150 neuropathologically confirmed control subjects from the UK and 133 belonging to the 1958 Wellcome cohort and did not find this variant. The Fisher exact test used to compare both allele and genotype frequencies between the clinically diagnosed PEP and the ESP cohort gave a two-tailed *p*-value of < 0.01 for allele and genotype frequencies. A search through two publically available databases (1000 genomes and National Institute of Environmental Health Sciences (NIEHS) Environmental Genome Project, Seattle, WA [URL: evs.gs.washington.edu/niehsExome/] [accessed on 03/2012]) revealed similarly low frequencies of the A152T variant (Supplementary Table 1) (1000 Genomes Project Consortium, 2010). This variant has been reported more frequently in Alzheimer's disease (AD) patients than in control subjects (Cruchaga et al., 2012).

#### 3.1.2 Case 1

##### 3.1.2.1 Clinical summary and neuropathology

The clinical and neuropathological features of this case were reported in a series of PEP (case 8) (Geddes et al., 1993). At age 26 years, this patient developed progressive levodopa-responsive parkinsonism with intact cognition. She had a long history of levodopa-induced orofacial dystonia. The disease duration was 54 years, and she died at age 80. A family history of dementia was noted but not elaborated in her medical records. In hindsight, the absence of encephalitis history would make the clinical diagnosis of PEP unlikely.

**Fig. 2:**
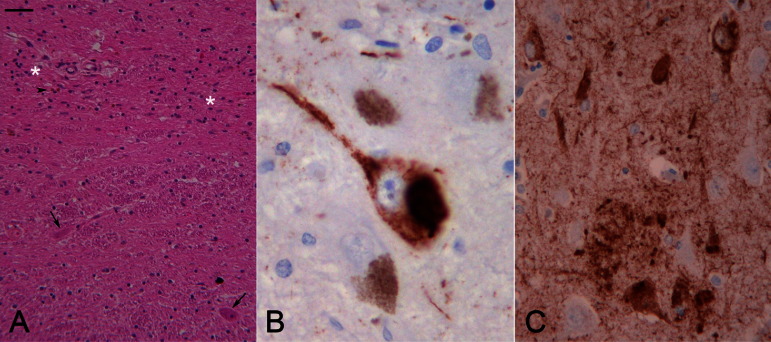
Case 1: Figure (A) shows severe loss of neuromelanin-containing neurons in the ventral tier of the substantia nigra (white asterisks), gliosis, and free pigment (arrow head). Non-pigmented neurons are still present in this region of the substantia nigra (arrows). Figure (B) shows scattered tau-positive NFTs in the nigra neurons. Figure (C) shows NFTs, NTs, and neuritic plaques in the CA1 hippocampal sub-region. (A—H&E, B and C—AT8 immunohistochemistry; bar on (A) represents 80 microns on A, 20 microns on B, and 40 microns on C). For interpretation of the references to color in this figure legend, the reader is referred to the Web version of this article.

Neuropathological review of this case has confirmed significant degree of neuronal loss in the substantia nigra, which was most severe in the ventrolateral tier. The tau pathology in the substantia nigra included neurofibrillary tangles (NFTs) and numerous, either fine or coarse, neuropil threads (NTs). Sparse NFTs and NTs were also seen in the midbrain tegmentum and, in addition, amyloid plaque-associated abnormal neurites were observed in the midbrain tectum. There were scattered NFTs and tau-positive threads in the striatum; occasional NFTs and threads were also seen in the globus pallidus and subthalamic nucleus. There was a single NFT with sparse NTs in the cerebellar dentate nucleus. There were amyloid-β (Aβ)-positive diffuse and mature plaques in the frontal, parietal, and temporal cortices. The tau pathology, which included classical NFTs, NTs, and plaque-associated neurites, was severe in the CA1 hippocampal subregion, entorhinal cortex, and fusiform gyrus, and it corresponded to Braak and Braak stage IV (Fig. 2). The tau inclusions were both 3R- and 4R-tau positive. Neither TDP-43-related pathology nor argyrophilic grain disease was observed.

After re-visiting the neuropathology of this case, our findings, as noted earlier, were not compatible with a pathological diagnosis of PEP. One might argue that the tau pathology, including the abundant NFTs and NTs in the substantia nigra, and the milder tau pathology in the subthalamic nucleus and globus pallidus can be entirely “age-related” (Mattila et al., 2002). In this scenario, the nigral cell loss would be unrelated to the nigral tau pathology, and the absence of α-synuclein pathology and negative genetics in the *PARK2* and *LRRK2* genes would indicate that an, as yet, unidentified neurodegenerative process was responsible for the underlying nigral cell loss. An alternative hypothesis is that the mild subcortical tau pathology with deposition of both 3R- and 4R-tau is related to the *MAPT* p.A152T mutation and an atypical tauopathy is responsible for the nigral cell loss.

#### 3.1.3 Case 2

##### 3.1.3.1 Clinical summary and neuropathology

When in her late 50s, this English woman began to develop a symmetrical akinetic rigid syndrome. Two years later, examination revealed hypomimia, drooling, predominant axial rigidity with mild symmetrical limb rigidity, brisk deep tendon reflexes, and flexor plantar response. A diagnosis of Parkinson's disease was made; however, there was no response to 700 mg/day of levodopa therapy. Her symptoms gradually deteriorated, and 7 years later, she developed dysarthria and dysphagia. In her mid 60s, she started to experience daily episodic dystonic spasms with an opisthotonus posture and oculogyric crisis. Examination in between these episodes revealed normal pursuit and saccadic eye movements, fixed retrocollis, slow tongue movement, anarthria, severe axial and limb rigidity, and dystonic posturing of the feet. Her cognitive function remained intact. The presence of oculogyric crisis prompted the neurologist to revise the diagnosis to PEP in the last year of her life. She died after disease duration of 10 years. However, in view of her lack of levodopa response, short disease duration, and the lack of encephalitis history, the clinical diagnosis of PEP would be very unlikely.

She has three younger sisters (Fig. 1). The second sister, now in her mid 80s, has developed dementia with short-term memory impairment, executive dysfunction, disorientation to time, and required assistance with dressing and housework. She has normal mobility. Her youngest sister, in her mid 70s, complains of forgetfulness but remains independent. These sisters were assessed in person and over the telephone, blind to the genetic analysis. Her third sister remains well. Both cognitively impaired sisters carried the mutation, whereas the healthy sister did not.

**Fig. 3:**
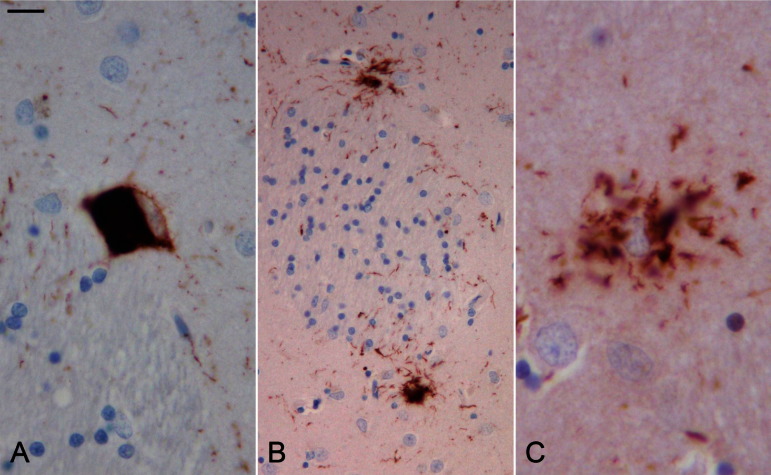
Case 2: Figure (A) shows a neuron with tau-positive NFT and fine NTs in the caudate. Figure (B) shows tufted astrocytes and NTs. Figure (C) shows tau-positive lesion containing 4-repeat tau isoform. (A and B—AT8 immunohistochemistry, C—4-repeat tau immunohistochemistry; bar on A represents 20 microns on A, 40 microns on figure B, and 15 microns on C). For interpretation of the references to color in this figure legend, the reader is referred to the Web version of this article.

Review of this case with immunohistochemistry using the AT8, 3R-tau, and 4R-tau antibodies indicated that the overall neuropathological findings fulfilled the diagnostic criteria for PSP (Ince et al., 2008; Litvan et al., 1996), as there were moderate numbers of AT8 and 4R-positive and 3R-negative tufted astrocytes, NFTs, NTs, and coiled bodies in the caudate, and similar, but milder, tau pathology was seen in the putamen. In addition, there was also severe gliosis and neuronal cell loss in the substantia nigra; significant reduction in size, nerve cell loss, and gliosis in the globus pallidus and subthalamic nucleus; but relatively good preservation of the cerebellar dentate nucleus and superior cerebellar peduncle. These findings, together with both neuronal and glial tau pathology in basal ganglia, brainstem, and cerebellar nuclei and relatively mild tau pathology in the cerebral cortex, pontine base, and cerebellar dentate nucleus, were in favor of the neuropathological diagnosis of the pallido-nigro-luysial atrophy variant of PSP (PSP-PNLA) (Ahmed et al., 2008). The age-related neurofibrillary pathology corresponded to Braak and Braak stage II; there was no evidence of argyrophilic grain disease (Fig. 3).

### 3.2 Secondary sequencing

After obtaining the findings outlined earlier in the article, we screened exon 7 of the *MAPT* gene in 114 PSP, 8 CBD, and 48 idiopathic Parkinson's disease cases from the QSBB archival collection. The mutation was identified in a case of CBD. More surprisingly, it was also found in one of the idiopathic Parkinson′s disease cases.

#### 3.2.1 Case 3

##### 3.2.1.1 Clinical summary and neuropathology

The CBD case developed symptoms in the mid-sixth decade, with early word finding difficulty and right-sided rigidity and gait difficulty evolving through nonfluent aphasia to mutism, increasing dependency, and death 11 years after symptom onset. There was no documented family history of dementing illness. The pathology included achromatic swollen neurons in the neocortex and limbic areas, numerous pretangles and NFTs, dense meshwork of tau-positive threads, astrocytic plaques in the frontal, temporal, and parietal cortices, and tau-positive threads and some coiled bodies in subcortical white matter. Additionally there were numerous TDP-43-positive neuronal cytoplasmic inclusions (NCIs) and neuronal intranuclear inclusions in globus pallidus, caudate, and putamen and severe cell loss in substantia nigra.

#### 3.2.2 Case 4

##### 3.2.2.1 Clinical summary and neuropathology

In his late 40s, this man developed an anxiety disorder and poor concentration, which resulted in him getting dismissed from his job. In the following year, he started to stutter and was diagnosed with depression. Two years later, he had memory loss, executive impairment, and slowness in movement. After the subsequent findings of markedly reduced tracer reuptake in the basal ganglia on [^123^I]FP-CIT DAT images, a diagnosis of Parkinson's disease with dementia was made when he was in his early 50s. He was commenced on levodopa therapy with moderate response. His cognitive function deteriorated markedly in the following year with fluctuating consciousness, disorientation, urinary incontinence, severe dementia, visual hallucination, myoclonus, and left-sided predominant akinetic rigidity. He died 7 years after the onset of his first symptoms.

Neuropathological findings included moderate to severe cell loss in the substantia nigra in association with α-synuclein-positive Lewy bodies and Lewy neurites. Frequent cortical Lewy bodies were observed in the frontal, temporal, parietal, cingulate gyrus, and transentorhinal cortices, corresponding to Braak stage 6 (Braak et al., 2003), diffuse neocortical Lewy body-type pathology according to consensus criteria (McKeith et al., 2005). NFTs were found in the hippocampus, entorhinal and transentorhinal cortices, which were consistent with Braak and Braak II. There were very few tau-positive NTs in the caudate nucleus, midbrain tegmentum, and periaqueductal gray. There was a single NFT in the locus coeruleus. A “low” level of Alzheimer disease pathologic change (A1, B1, C0) was identified (Hyman et al., 2012). The neuropathological diagnoses were Parkinson's disease with dementia and pathological aging.

## 4 Discussion

Our intention in this study was to try and understand whether there were genetic factors in the *MAPT* gene that contributed to those cases with complex tauopathies, previously often diagnosed as PEP. Our finding of the p.A152T *MAPT* mutation in two of eight of these cases, together with the previous report from Kovacs et al. (2011) with the limited segregation in the family of proband (case 1), together with its absence from the control subjects we sequenced and its rarity in public databases, supports the view that this variant contributes to disease risk. The finding that the mutation occurs in one of eight cases of CBD could also be adduced to support this view.

However, the identification of the variant in a case of idiopathic PD was a surprise, even though this case is unusual for such a young case in having a considerable burden of tau pathology. Additionally, the disease picture in all the cases we report varies both clinically and pathologically, and although we show some evidence for segregation in the single family in which this analysis was possible, in most cases family history was not reported and, unlike in classic cases with *MAPT* mutations, the tau pathology was very varied, both in morphology and distribution: being 4 repeat in cases 2 and 3 and a classic mixture of 3 and 4 repeat in cases 1 and 4.

The position of the variant is interesting in that it creates an additional phosphorylation site in the tau protein, and tau hyperphosphorylation has been repeatedly suggested to contribute to tangle formation and cell death sites (Rademakers et al., 2004). One plausible suggestion is that this mutation contributes to disease pathogenesis through the creation of an additional phosphorylation site, but this is not sufficient, of itself, to cause disease in the way that the *MAPT* splice and P301L mutations do. The mutated residue is next to Thr153 (T153), a phosphorylation site which is a target for proline-directed kinases such as mitogen-activated protein kinase (MAPK), cell division protein kinase 5 (cdk5), and glycogen synthase kinase-3 alpha (GSK3α). Using an antibody against the phosphorylated T153 (pT153), Augustinack et al. (2002) showed that although intraneuronal and extraneuronal tangles were labeled, the dominant staining was punctate and of pretangles within morphologically intact neurons, with normal cellular integrity and well-preserved dendrites. A similar staining pattern of pretangles was observed with the conformation-specific TG3 antibody (Jicha et al., 1997) and a phospho-tau-specific pS262 antibody, and they suggest that these antibodies label an early stage of tangle formation characterized by punctate inclusions that precede fully fledged fibrillar tangles (Augustinack et al., 2002). It is possible that the additional Thr152 increases phosphorylation and thus further contributes to the early stages of the fibrillogenic pathway.

*LRRK2* mutations offer a precedent for this complex relationship between mutations and disease. Some mutations (e.g., p.G2019S) cause disease in an almost fully penetrant autosomal dominant fashion (Paisán-Ruíz et al., 2004), whereas others (e.g., p.G2385R) increase disease risk by a modest amount (Tan et al., 2010). Additionally, and also similarly to the situation with regard to the p.A152T *MAPT* variant, *LRRK2* mutations cause variable pathology, usually Lewy body pathology, but sometimes tangle or TDP-43 pathology (Zimprich et al., 2004). For the true role of this variant in neurological disease to be determined, clearly very large numbers (thousands) of cases with various tauopathies and control subjects will need to be sequenced, and certainly until that time, these data would not support this mutation becoming part of the clinical test in the way that the penetrant mutations are.

These data have also caused us to re-examine our views on PEP. PEP has now become a historical illness with an exception of a few sporadic cases. In the past, there was a high prevalence rate of PEP after the simultaneous outbreak of encephalitis lethargica (von Economo's encephalitis or “sleepy sickness” that was portrayed in the movie “Awakenings”) and H1N1 influenza A virus pandemics in Europe from the late 1910s until the mid-1920s (Economo, 1931). It has been speculated, but never proven, that the influenza A virus caused the encephalitis, which in turn led to a parkinsonian disorder in a substantial number of sufferers (Calne and Lees, 1988). However, neither of our primary patients had a clear history of encephalitis or a flu-like illness preceding the development of parkinsonian features, which makes the clear diagnosis of PEP doubtful, especially in view of the genetic findings we report. PEP, like amyotrophic lateral sclerosis/parkinsonism-dementia comples of Guam (ALS/PDC) complex of Guam (Geddes et al., 1993), has been a prevalent tauopathy, which appeared and then disappeared without any clear explanation being found (Steele, 2005). In both these cases and in the smaller tauopathy outbreaks on the Kii peninsular and on St Martinique (Caparros-Lefebvre et al., 2006), our understanding of these diseases has been limited by the collection and survival of too few samples for modern analytical investigations.

## 5 Conclusions

We conclude that the rare p.A152T variant is likely to increase the susceptibility to the development of neurodegenerative conditions with abnormal tau accumulation. Further functional studies are necessary to fully dissect the functional consequences and precise pathogenic mechanisms associated with this mutation. The evidence for the pathogenicity of this variant is not strong enough for these data to be used in genetic counseling.

## Disclosure statement

None of the authors have potential or actual conflicts of interest, and all the authors have seen the manuscript before submission.

The work was funded by the Reta Lila Weston Foundation, the PSP Brain Bank, USA Parkinson's Disease Foundation and the MRC/Wellcome Trust through a PD Centre Grant, and by the Wellcome Trust. The funding source had no role in study design, data collection and analysis, decision to publish, or preparation of the manuscript.

There is no animal work in the manuscript, and the human work on blood and pathology materials has been carried out in compliance with UK regulations.
