# Supplementary material for: The MAPT p.A152T variant is a risk factor associated with tauopathies with atypical clinical and neuropathological features
Source: Neurobiol Aging. 2012 Sep;33(9):2231.e7–2231.e14. doi: 10.1016/j.neurobiolaging.2012.04.006 (PMC3657164; doi:10.1016/j.neurobiolaging.2012.04.006)
Supplement: Supplementary Table 1 [file mmc1.docx]

| Database | Ethnicity of subjects | Total number of subjects included | Genotype frequency of heterozygous carriers of the A152T *MAPT* variant | Genotype frequency of homozygous carriers of the A152T *MAPT* variant | Allele frequency of the c.G454A variant |
| --- | --- | --- | --- | --- | --- |
| NHLBI GO Exome Sequencing Project (ESP) | European American | 3510 | 19/3510 (0.54%) | 0/3510 (0%) | 19/7020 |
| NHLBI GO Exome Sequencing Project (ESP) | African American | 1869 | 3/1869 (0.0016%) | 0/1869 (0%) | 3/3738 |
| 1000 genomes | Ad Mixed American*^3^ | 181 | 1/181 (0.0055%) | 0/181 (0%) | 1/362 |
| 1000 genomes | European*^4^ | 381 | 3/381 (0.0079%) | 0/381 (0%) | 3/762 |
| 1000 genomes | African*, East Asian*^1^  South Asian*^2^ | 532 | 0/532 (0%) | 0/532 (0%) | 0/1064 |
| NIEHS | Caucasian | 22 | 0/22 (0%) | 0/22 (0%) | 0/44 |
| NIEHS | African American | 14 | 0/14 (0%) | 0/14 (0%) | 0/28 |
| NIEHS | Asian | 24 | 0/24 (0%) | 0/24 (0%) | 0/48 |
| NIEHS | Hispanic | 22 | 0/22 (0%) | 0/22 (0%) | 0/44 |
| NIEHS | Yoruban | 13 | 0/13 (0%) | 0/13 (0%) | 0/26 |

* Yoruba in Ibadan, Nigera; Luhya in Webuye, Kenya; Gambian in Western Divisons in The Gambia; Mende in Sierra Leone; Esan in Nigera; American's of African Ancestry in SW USA; African Carribean in Barbados

*^1^ Han Chinese in Bejing, China; Japanese in Tokyo, Japan; Southern Han Chinese; Chinese Dai in Xishuanagbanna, China; Kinh in Ho Chi Minh City, Vietnam

*^2^ Gujarati Indian from Houston, Texas; Punjabi from Lahore, Pakistan; Bengali from Bangladesh; Sri Lankan Tamil from the UK; Indian Telugu from the UK

*^3^ Mexican Ancestry from Los Angeles USA; Puerto Rican from Puerto Rica; Colombian from Medellian, Colombia; Peruvian from Lima, Peru

*^4^ Utah Residents (CEPH) with Northern and Western European ancestry; Toscani in Italia; Finnish in Finland; British in England and Scotland; Iberian population in Spain
